# Supplementary material for: Post-Transcriptional Control of Mating-Type Gene Expression during Gametogenesis in Saccharomyces cerevisiae
Source: Biomolecules. 2021 Aug 17;11(8):1223. doi: 10.3390/biom11081223 (PMC8394074; doi:10.3390/biom11081223)
Supplement: Supplementary file 1 [file biomolecules-11-01223-s001.zip › biomolecules-1322428-supplementary/Supplementary Figures.pdf]

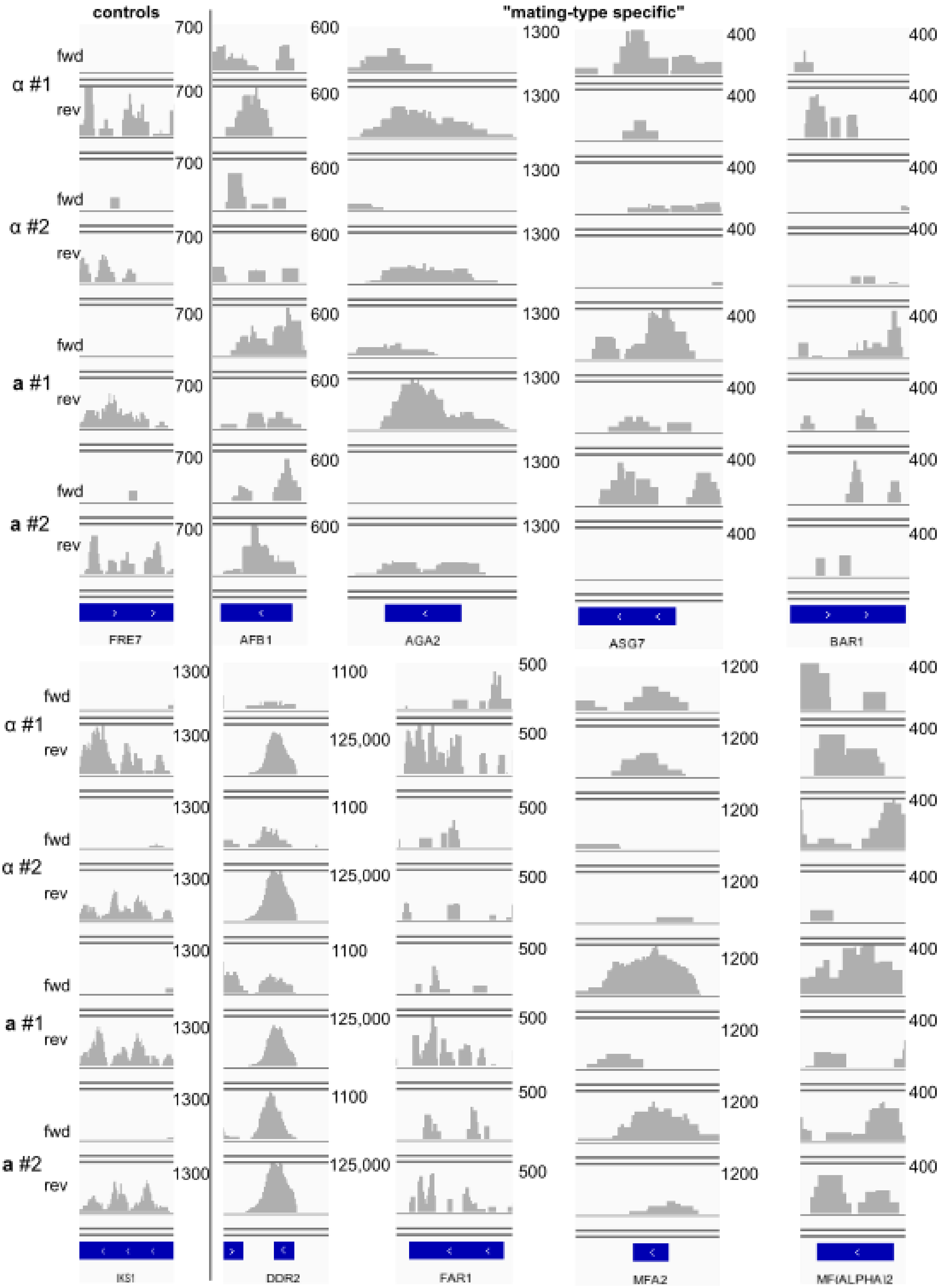
controls
"mating-type specific"
α #1
α #2
a #1
a #2
fwd
rev
700
600
1300
400
1100
125,000
500
1200
FRE7
AFB1
AGA2
ASG7
BAR1
IKS1
DDR2
FAR1
MFA2
MF(ALPHA)2

Figure S1. Spores harbor antisense transcripts to many “mating-type-specific” genes. RNA-seq reads from spores sorted by mating type were mapped onto the genome in a strand-specific manner. Biological replicates are numbered as “#1” and “#2”. For all but *DDR2*, the *y* axis scale is the same for both strands, as indicated by the numbers at top right of each plot.

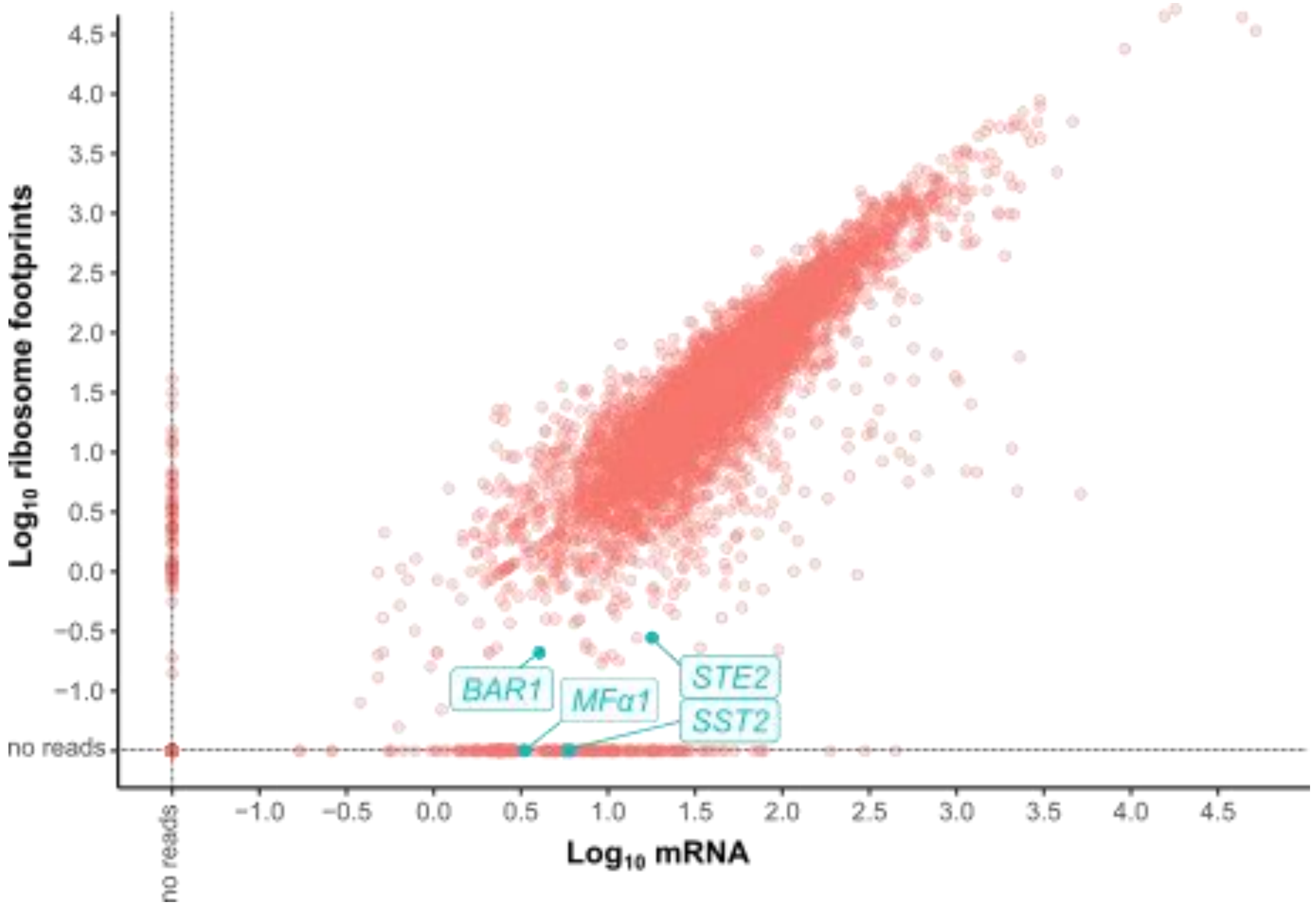

Figure S2. Limited translation in spores of mating-type-specific transcripts. Published transcriptome-wide RNA-seq and ribosome profiling data [6] from the final (18-hour) time point of synchronous sporulation using the traditional synchronization method were retrieved from the GEO database (GSM843742 and GSM843775) and plotted against each other after taking the $\log_{10}$ value for each transcript. For values that were 0, the $\log_{10}$ value is undefined, so these transcripts were assigned values of -1.5 for plotting purposes, and are marked here as “no reads”.

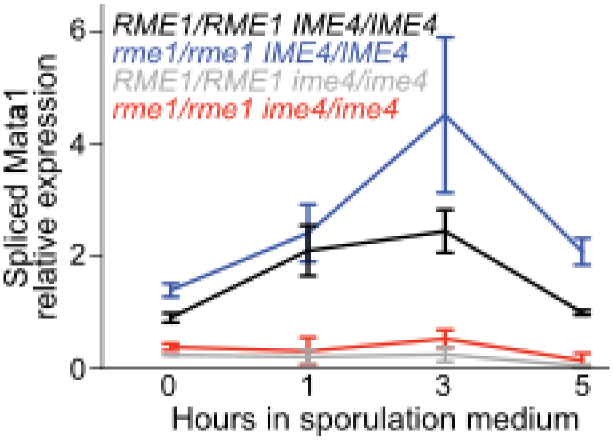

Figure S3. m^{6}A methylation promotes Mata1 induction during sporulation. Quantitative RT-PCR was used to quantify at each time point the amount of spliced Mat**a**1 relative to Act1. Ratios were normalized to *RME1/RME1 IME4/IME4* at time point 0. "*RME1*" refers to the ins-308A insertion polymorphism in the *RME1* upstream region. "*rme1*' refers to the allele found in S288C-background strains that increases *RME1* expression [15]. "*RME1/RME1 IME4/IME4*" and "*RME1/RME1 ime4/ime4*" are the same strains as in Figure 3a. "*rme1/rme1 IME4/IME4*" and "*rme1/rme1 ime4/ime4*" are strains GBy222 and GBy225. Plot symbols show means and standard deviations from three biological replicates.
